# Fumagillin Shortage: How to Treat *Enterocytozoon bieneusi* Microsporidiosis in Solid Organ Transplant Recipients in 2024?

**DOI:** 10.3389/ti.2024.13518

**Published:** 2024-12-12

**Authors:** Cyril Garrouste, Philippe Poirier, Charlotte Uro-Coste, Xavier Iriart, Nassim Kamar, Julie Bonhomme, Eve Calvar, Solène Le Gal, Luca Lanfranco, Brice Autier, Lucien Rakoff, Marie-Fleur Durieux, Clément Danthu, Florent Morio, Clément Deltombe, Alicia Moreno-Sabater, Nacera Ouali, Damien Costa, Dominique Bertrand, Adélaïde Chesnay, Philippe Gatault, Meja Rabodonirina, Emmanuel Morelon, Jérôme Dumortier, Emilie Sitterlé, Anne Scemla, Samia Hamane, Laurène Cachera, Céline Damiani, Coralie Poulain, Coralie L’Ollivier, Valérie Moal, Laurence Delhaes, Hannah Kaminski, Estelle Cateau, Laure Ecotière, Julie Brunet, Sophie Caillard, Stéphane Valot, Claire Tinel, Nicolas Argy, Quentin Raimbourg, Marie Gladys Robert, Johan Noble, Aude Boignard, Françoise Botterel, Marie Matignon, Anne-Pauline Bellanger, Thomas Crépin, Jordan Leroy, Arnaud Lionet, Anne Debourgogne, Muriel Nicolas, Joëlle Claudéon, Maxime Moniot, Céline Lambert, Céline Nourrisson

**Affiliations:** ^1^ Service de Néphrologie, CHU Clermont-Ferrand, Clermont-Ferrand, France; ^2^ Service de Parasitologie-Mycologie, 3IHP, Inserm U1071, M2iSH, USC-INRAE 1382, Université Clermont Auvergne, CHU Clermont-Ferrand, Clermont-Ferrand, France; ^3^ Centre National de Référence des Cryptosporidioses, Microsporidies et Autres Protozooses Digestives, Laboratoire Associé de Clermont-Ferrand, Clermont-Ferrand, France; ^4^ Service de Parasitologie-Mycologie, Institut Toulousain des Maladies Infectieuses et Inflammatoires (Infinity), CNRS UMR5051, INSERM UMR1291, Université Toulouse III Paul Sabatier, CHU Toulouse, Toulouse, France; ^5^ Service de Néphrologie, CHU Toulouse, Toulouse, France; ^6^ Service de Parasitologie-Mycologie, ToxEMAC-ABTE, Université de Normandie Unicaen, CHU Caen, Caen, France; ^7^ Service de Néphrologie, CHU Caen, Caen, France; ^8^ Service de Parasitologie-Mycologie, CHU Brest, Brest, France; ^9^ Service de Néphrologie, CHU Brest, Brest, France; ^10^ Service de Parasitologie-Mycologie, CHU Rennes, Rennes, France; ^11^ Service de Néphrologie, CHU Rennes, Rennes, France; ^12^ Service de Parasitologie-Mycologie, CHU Limoges, Limoges, France; ^13^ Service de Néphrologie, CHU Limoges, Limoges, France; ^14^ Cibles et Médicaments des Infections et de l’Immunité, Nantes Université, CHU Nantes, Nantes, France; ^15^ Service de Néphrologie, CHU Nantes, Nantes, France; ^16^ Service de Parasitologie-Mycologie, Centre d’Immunologie et de Maladies Infectieuses (Cimi-Paris), Inserm U1135, Sorbonne Université, AP-HP, Hôpital Saint-Antoine, Paris, France; ^17^ Service de Néphrologie, Assistance Publique-Hôpitaux de Paris, Hôpital Saint-Antoine, Paris, France; ^18^ Parasitology-Mycology laboratory, EA 7510 ESCAPE Epidemiosurveillance and Circulation of Parasites in the Environment, University of Rouen Normandie, University Hospital of Rouen, National Reference Center (NRC) for cryptosporidiosis, microsporidia and other digestive protozoa, Rouen, France; ^19^ Service de Néphrologie, CHU Rouen, Rouen, France; ^20^ Service de Parasitologie-Mycologie, CHU Tours, Tours, France; ^21^ Service de Néphrologie, CHU Tours, Tours, France; ^22^ Service de Parasitologie-Mycologie, Hospices Civils de Lyon, Lyon, France; ^23^ Service de Néphrologie, Hospices Civils de Lyon, Lyon, France; ^24^ Service d’Hépato-Gastroentérologie, Hospices Civils de Lyon, Hôpital Edouard Herriot, Université Claude Bernard Lyon 1, Lyon, France; ^25^ Service de Parasitologie-Mycologie, Assistance Publique-Hôpitaux de Paris, Hôpital Necker, Paris, France; ^26^ Service de Néphrologie, Assistance Publique-Hôpitaux de Paris, Hôpital Necker, Paris, France; ^27^ Service de Parasitologie-Mycologie, Assistance Publique-Hôpitaux de Paris, Hôpital Saint-Louis, Paris, France; ^28^ Service de Néphrologie, Assistance Publique-Hôpitaux de Paris, Hôpital Saint-Louis, Paris, France; ^29^ Service de Parasitologie et Mycologie Médicales et Agents Infectieux, Résistance et Chimiothérapie (AGIR), UR 4294, CHU Amiens-Picardie, Université de Picardie Jules Verne, Amiens, France; ^30^ Service de Néphrologie, CHU Amiens, Amiens, France; ^31^ Service de Parasitologie-Mycologie, Assistance Publique-Hôpitaux de Marseille, IHU Méditerranée Infection, Marseille, France; ^32^ Service de Néphrologie, Assistance Publique-Hôpitaux de Marseille, Paris, France; ^33^ Service de Parasitologie-Mycologie, CHU Bordeaux, Bordeaux, France; ^34^ Service de Néphrologie-Transplantation-Dialyse-Aphéréses, CHU Bordeaux, Bordeaux, France; ^35^ Service de Parasitologie-Mycologie, CHU Poitiers, Poitiers, France; ^36^ Service de Néphrologie, CHU Poitiers, Poitiers, France; ^37^ Service de Parasitologie-Mycologie, CHU Strasbourg, Strasbourg, France; ^38^ Service de Néphrologie, CHU Strasbourg, Strasbourg, France; ^39^ Service de Parasitologie-Mycologie, CHU Dijon, Dijon, France; ^40^ Service de Néphrologie, CHU Dijon, Dijon, France; ^41^ Service de Parasitologie-Mycologie, Assistance Publique-Hôpitaux de Paris, Hôpital Bichat, Paris, France; ^42^ Service de Néphrologie, Assistance Publique-Hôpitaux de Paris, Hôpital Bichat, Paris, France; ^43^ Service de Parasitologie-Mycologie, Université Grenoble Alpes, CHU Grenoble Alpes, Grenoble, France; ^44^ Service de Néphrologie, CHU Grenoble, Grenoble, France; ^45^ Service de Cardiologie, CHU Grenoble, Grenoble, France; ^46^ Service de Parasitologie-Mycologie, Assistance Publique-Hôpitaux de Paris, Hôpital Henri Mondor, Paris, France; ^47^ Service de Néphrologie, Assistance Publique-Hôpitaux de Paris, Hôpital Henri Mondor, Paris, France; ^48^ Service de Parasitologie-Mycologie, CHU Besançon, Besançon, France; ^49^ Service de Néphrologie, CHU Besançon, Besançon, France; ^50^ Service de Parasitologie-Mycologie, CHU Lille, Lille, France; ^51^ Service de Néphrologie, CHU Lille, Hôpital Huriez, Lille, France; ^52^ Service de Parasitologie-Mycologie, CHU Nancy, Nancy, France; ^53^ Service de Parasitologie-Mycologie, CHU Pointe-à-Pitre, Guadeloupe, France; ^54^ Service de Néphrologie, CHU Pointe-à-Pitre, Guadeloupe, France; ^55^ Unité de Biostatistiques, DRCI, CHU Clermont-Ferrand, Clermont-Ferrand, France

**Keywords:** solid organ transplant, nitazoxanide, microsporidiosis, Enterocytozoon bieneusi, infectious diarrhea

## Abstract

Intestinal microsporidiosis caused by *Enterocytozoon bieneusi* is an opportunistic infection that especially affects solid organ transplant (SOT) recipients. Management revolves around tapering the immunosuppressive regimen and/or using a specific anti-microsporidia treatment, but only fumagillin has demonstrated efficacy for treatment of this infection. Since fumagillin has been commercially discontinued, nitazoxanide is increasingly being used in this indication. We aimed to describe therapeutic management of *E. bieneusi* infections in this context. We conducted a French nationwide observational retrospective study on reported cases of *E. bieneusi* infections in SOT recipients. We identified 154 cases: 64 (41.6%) were managed by simply modifying the immunosuppressive regimen, 54 (35.1%) were given fumagillin, and 36 (23.4%) were given nitazoxanide. Clinical remission rate ranged from 77.8% to 90.7% and was not significantly different between therapeutic strategies but tended to be lower with nitazoxanide. Stool negativization rate was highest with fumagillin (91.7%) and lowest with nitazoxanide (28.6%). Relapses occurred in 6.9% of cases and were more frequent with nitazoxanide (14.3%). This study shows that tapering immunosuppression can result in a satisfactory remission rate but is sometimes accompanied by relapses. Nitazoxanide had limited effectiveness, whereas fumagillin had good results that provide a solid rationale for bringing fumagillin back to market.

**Trial Registration Number:**

ClinicalTrials.gov ID: NCT05417815.

## Graphical Abstract

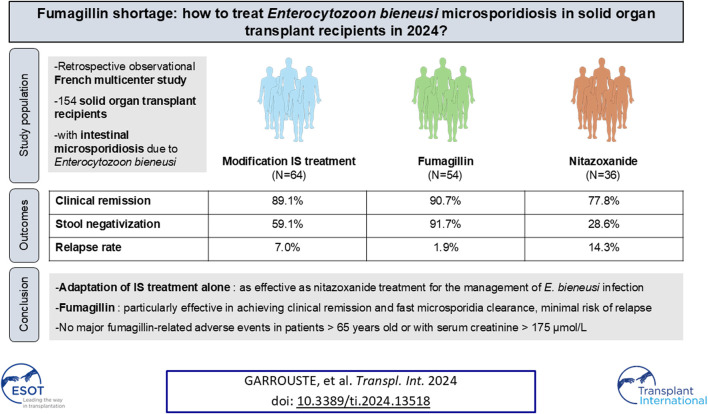

## Introduction

*Enterocytozoon bieneusi* is by far the most common microsporidia species causing intestinal microsporidiosis, which manifests as profuse watery diarrhea and abdominal pain [1]. *Enterocytozoon bieneusi* microsporidiosis can occur in both immunocompetent and immunocompromised individuals, but significantly affects immunocompromised solid organ transplant (SOT) recipients [2, 3]. While in the immunocompetent, the infection will generally result in acute diarrhea, in the immunocompromised, it will become chronic, which can lead to significant weight loss and dehydration.

Management of these infections in SOT patients is not fully standardized but involves tapering immunosuppression, possibly also associated with specific treatment. Only fumagillin, a mycotoxin produced by the fungus *Aspergillus fumigatus*, has demonstrated effectiveness against *E. bieneusi* infections in clinical trials [4, 5]. Serious adverse events were observed in 25% of patients and especially included dose-related hematologic toxicity that manifested as thrombocytopenia and/or neutropenia, requiring hospitalization for the duration of treatment [6]. Fumagillin has been out of stock on several occasions in the past and has not been commercialized since 2019, in some cases leading to therapeutic impasse. In the absence of fumagillin, the proposed alternative is nitazoxanide, a broad-spectrum antiparasitic drug effective against a broad range of protozoan and helminthic infections. However, the effectiveness of nitazoxanide against microsporidia has never been formally evaluated, but some successes have been described in case reports [7–11]. Nitazoxanide has few reported serious side effects, but there have been occasional reports of nitazoxanide-induced liver injury [12].

Given this context of difficulty treating intestinal microsporidioses, the French National Reference Center for microsporidioses is regularly asked to advise on therapeutic options for SOT patients. Here, to address this need, we worked with the French network of transplant recipients to conduct a retrospective observational study of *E. bieneusi* infections and therapeutic management in a French cohort of SOT patients.

## Patients and Methods

### Ethical Statement

This retrospective study was approved by our local research ethics committee (“Comité de Protection des Personnes Sud-Est VI,” France) and performed in compliance with French data privacy policy (approval #MPP220505).

### Study Population

We used the French National Reference Center (NRC) for Microsporidiosis, the Spiesser and Divat groups, and the “Groupe français de recherche en greffe de foie” to retrospectively collect microsporidiosis cases in adult SOT recipients in France between 2018 and 2021 where *E. bieneusi* was identified as the causal agent. Briefly, since 2018 and on a voluntary basis, French laboratories diagnosing a case of microsporidiosis report their case to the NRC (by sending samples and providing clinical data). The network of participating laboratories is sufficiently extensive to allow representation of the entire French territory (mainland and overseas). The NRC case register was used to identify centers to contact for the present study. At the same time, two French transplant medicine networks, Spiesser and Divat, and the “Groupe français de recherche en greffe de foie”, were also contacted to identify cases corresponding to the study inclusion criteria. So these two approaches allowed to identify as many cases of microsporidiosis as possible. As fumagillin production was discontinued in 2019, the number of patients treated with fumagillin over the 2018–2021 period was low compared to other management strategies. We therefore also included fumagillin-treated patients from the TRANS-SPORE registry, a previous retrospective observational study on microsporidiosis in French kidney transplant recipients for the period 2005–2017 carried out in six university hospital centers [2]. Cases were defined by the presence of persistent diarrhea (*i.e.*, ≥3 liquid stools per day for more than 2 weeks) and detection of microsporidia spores by microscopic examination of fecal smears (after Van Gool chemiluminescent staining, Weber’s modified trichrome staining, or immunofluorescent staining) and/or molecular methods. Identification to species level was achieved using species-specific antibodies or PCR. Exclusion criteria were age <18 years, extraintestinal microsporidiosis, and microsporidiosis caused by a species other than *E. bieneusi*.

Patient demographics and medical records were retrieved from the hospital registries, and the following data were recorded: age, gender, type of organ transplant, retransplantations, date of current transplant, clinical presentation associated with microsporidiosis, values of biological parameters at diagnosis (hemoglobin, lymphocyte and CD4 counts, neutrophil count, platelet count, C-reactive protein (CRP), serum creatinine, residual concentrations of immunosuppressive drugs before and at diagnosis) and after treatment (platelet count, serum creatinine), microsporidiosis treatment, associated infections, relapses, and graft and patient outcomes at 1 year.

### Definitions of Groups

The included patients were divided into three groups according to therapeutic management. (i) Patients who did not receive any specific drug against *E. bieneusi* and who were managed with a modification of immunosuppressive (IS) treatment that could be carried out in a context of a too high IS trough levels at diagnosis of microsporidiosis or in a context of ‘normal’ trough levels per standard-of-care [13, 14], belonged to the “MIT” group. (ii) Patients who received fumagillin, with or without modification of the IS treatment, were included in the “FUM” group. (iii) Patients who received nitazoxanide, with or without modification of the IS treatment, belonged to the “NTZ” group. Patients who received one treatment then the other were classified under their second treatment group (considering that the first had not been effective).

Three criteria were studied to evaluate the effectiveness of therapeutic management: (i) the resolution of clinical manifestations (*i.e.*, resolution of diarrhea) at the end of the treatment/modification of the IS treatment, (ii) the stool negativization (*i.e.*, stool clearance), determined by PCR during and/or after treatment, and (iii) the clinical relapse (reappearance of diarrhea) rate.

### Statistical Analysis

No sample size estimation was performed. As this is a rare disease, we aimed for the exhaustiveness. Statistical analysis was performed using Stata software (version 15; StataCorp, College Station, TX). All tests were two-sided, with an alpha level set at 5%. Categorical data are reported as number of patients and percentages, and quantitative data are reported as mean ± standard deviation or median [25th; 75th percentiles]. Baseline between-groups comparisons were performed using a Chi-squared test or Fisher’s exact test for categorical data, and ANOVA or a Kruskal-Wallis test for quantitative data. Between-groups comparisons on outcomes were performed using linear or generalized linear mixed models, with hospital center as random effect. In particular, two multivariable analyses were performed on clinical remission using a mixed effects logistic regression. In these analyses, the fixed effects were: group, age, sex, and serum creatinine at diagnosis, in the first model; and group, age, sex, and renal failure, in the second model. Finally, time to stool negativization and time to clinical remission (censored data) were estimated by the Kaplan-Meier method and compared between groups using the log-rank test.

## Results

### Study Population

The characteristics of the patient cohort are given in Table 1. A total of 154 patients from 26 French hospital centers were included. They were mainly males (55.8%), aged 56.2 ± 14.5 years, and the vast majority kidney transplant recipients (93.5%).

**TABLE 1:** Clinical characteristics of the patients.

	All patients (n = 154)	MIT (n = 64, 41.6%)	Fumagillin (n = 54, 35.1%)	Nitazoxanide (n = 36, 23.4%)	p
Age at diarrhea onset (years) (n = 145)	56.2 ± 14.5	53.6 ± 15.0	56.6 ± 15.3	60.5 ± 11.4	0.09
Male sex	86 (55.8)	31 (48.4)	35 (64.8)	20 (55.6)	0.20
Previous transplant	26 (16.9)	8 (12.5)	11 (20.4)	7 (19.4)	0.47
Transplant(s)					
Kidney	144 (93.5)	59 (92.2)	51 (94.4)	34 (94.4)	0.92
Heart	8 (5.2)	3 (4.7)	1 (1.9)	4 (11.1)	0.17
Liver	6 (3.9)	3 (4.7)	3 (5.6)	0 (0.0)	0.50
Lung	1 (0.6)	1 (1.6)	0 (0.0)	0 (0.0)	1.00
Pancreas	3 (1.9)	2 (3.1)	0 (0.0)	1 (2.8)	0.46
Multi-organs transplant	7 (4.5)	3 (4.7)	1 (1.9)	3 (8.3)	0.43
Baseline serum creatinine (µmol/L) (n = 101)	142 [112; 180]	135 [110; 175]	146 [112; 200]	146 [127; 175]	0.68
Time between last transplantation and first positive PCR (years) (n = 151)	6 [2; 10]	6 [2; 10]	6 [3; 11]	5 [3; 10]	0.98

Data are presented as number of patients (percentages), mean ± standard deviation, or median [25th; 75th percentiles]. Comparisons between groups were made with Chi-squared test or Fisher’s exact test for categorical data, and with ANOVA, or Kruskal-Wallis test for quantitative data. In the first column, “n” is the number of available data when there is missing data. MIT: modification of immunosuppressive treatment; PCR: polymerase chain reaction.

The MIT group included 64 patients (41.6%), the FUM group included 54 patients (35.1%) and the NTZ group included 36 patients (23.4%) (Table 1).

There were 27 patients treated with fumagillin from the TRANS-SPORE registry [2] and 27 from the French National Reference Center for microsporidiosis for the 2018–2021 period. There were no differences between these two subcohorts on any of the criteria tested, *i.e.*, clinical characteristics (age, gender, transplant, time to onset of microsporidiosis), clinical remission rate, creatinine at 3 months, relapse, organ failure, and death (data not shown).

### Clinical and Biological Presentation at Microsporidiosis Diagnosis

**FIGURE 1:**
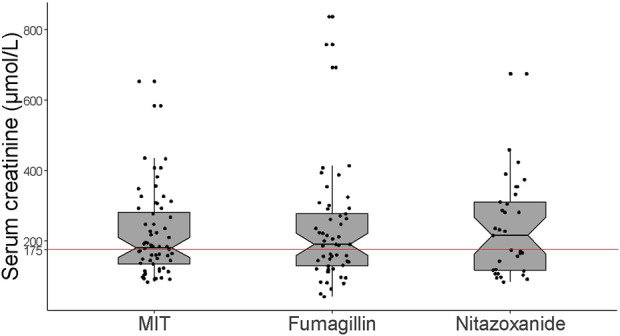
Serum creatinine values at microsporidiosis diagnosis according to therapeutic management group. The red line at 175 μmol/L represents the maximum creatinine value beyond which fumagillin is contraindicated according to the safety notice. MIT: modification of immunosuppressive treatment.

Microsporidiosis onsetted at a median time of 5.6 [2.3; 9.7] years following transplantation (Table 1). In addition to diarrhea, more than half (53.9%) of the patients presented weight loss (approximately 8% [5; 12] of ideal weight) (Table 2). The other reported symptoms were nausea/vomiting (37.7%), asthenia (20.8%), abdominal pain (20.1%), fever (5.2%), anorexia (3.9%), dehydration (3.2%), bloating (1.9%), hypotension (1.3%), and dysphagia (1.3%). There were no between-group differences in these symptoms except for abdominal pain (*p* = 0.045, Table 2). Microsporidia diagnosis was performed with a median delay of 19 [12; 39] days after the onset of diarrhea. At diagnosis, median values of hematological and inflammatory parameters were normal, except for lymphocyte count which was decreased (Table 2). More than half of the patients (n = 55/100, 55.0%) had acute renal failure according to the acute kidney injury network (AKIN) classification [15], of which 34 (34.0%) had AKIN 2 or 3 renal failure (Table 2). Overall, median serum creatinine value was 187 [124; 293] µmol/L at diagnosis. Note that even though fumagillin is contraindicated for creatinine values above 175 μmol/L, median serum creatinine value in the FUM group was 191 [129; 278] µmol/L (Figure 1). In the MIT group, 51.9% of patients (n = 14/27) had AKIN 1 renal failure, whereas 50.0% of patients (n = 7/14) in both the FUM and NTZ groups had AKIN 3 renal failure. Nearly a third of patients (30.5%) had another concurrent infection (Supplementary Table S1).

**TABLE 2:** Clinical presentation of microsporidiosis and laboratory characteristics at diagnosis.

	All patients (n = 154)	MIT (n = 64)	Fumagillin (n = 54)	Nitazoxanide (n = 36)	p
Symptoms					
Diarrhea	154 (100)	64 (100)	54 (100)	36 (100)	NA
Fever (>38.5°C)	8 (5.2)	5 (7.8)	1 (1.9)	2 (5.6)	0.41
Nausea/vomiting	58 (37.7)	24 (37.5)	18 (33.3)	16 (44.4)	0.57
Asthenia	32 (20.8)	15 (23.4)	10 (18.5)	7 (19.4)	0.79
Abdominal pain	31 (20.1)	16 (25.0)	5 (9.3)	10 (27.8)	0.045
Weight loss	83 (53.9)	29 (45.3)	33 (61.1)	21 (58.3)	0.19
Weight loss (%) (n = 75)	8 [5; 12]	7 [5; 13]	9 [5; 11]	6 [5; 11]	0.71
Hospitalization rate	127 (82.5)	47 (73.4)	50 (92.6)	30 (83.3)	0.02
Serum creatinine (µmol/L) (n = 150)	187 [124; 293] (43; 838)	182 [136; 285] (85; 653)	191 [129; 278] (43; 838)	216 [118; 311] (85; 676)	0.88
Renal failure	55/100 (55.0)	27/49 (55.1)	14/23 (60.9)	14/28 (50.0)	0.74
AKIN stage					0.22
1	21/55 (38.2)	14/27 (51.9)	3/14 (21.4)	4/14 (28.6)	
2	14/55 (25.4)	7/27 (25.9)	4/14 (28.6)	3/14 (21.4)	
3	20/55 (36.4)	6/27 (22.2)	7/14 (50.0)	7/14 (50.0)	
CRP (mg/L) (n = 115)	4 [1; 11]	3 [1; 11]	5 [2; 15]	3 [1; 8]	0.31
Hemoglobin (g/dL) (n = 150)	11.6 [10.1; 12.9]	11.4 [10.0; 12.4]	11.7 [10.3; 13.4]	11.9 [10.1; 12.9]	0.87
Platelets (G/L) (n = 133)	226 [169; 268]	236 [168; 279]	219 [165; 264]	217 [173; 263]	0.96
PNN (G/L) (n = 133)	4.7 [3.3; 6.6]	4.7 [3.1; 7.1]	5.3 [3.3; 8.1]	4.5 [3.3; 5.8]	0.33
Lymphocyte (G/L) (n = 130)	1.08 [0.64; 1.60]	1.19 [0.77; 1.82]	1.10 [0.64; 1.81]	1.01 [0.51; 1.30]	0.08

Data are presented as number of patients (percentages), or median [25th; 75th percentiles] (range). Comparisons between groups were made with Chi-squared test or Fisher’s exact test for categorical data, and with Kruskal-Wallis test for quantitative data. In the first column, “n” is the number of available data when there is missing data. AKIN: acute kidney injury network (score 3 is more serious than score 1); CRP: C-reactive protein; MIT: modification of immunosuppressive treatment; NA: not applicable; PNN: polynuclear neutrophil.

### Therapeutic Management

A high proportion of patients were hospitalized (82.5%), particularly patients receiving fumagillin (92.6% vs. 73.4% in the MIT group vs. 83.3% in the NTZ group, *p* = 0.02) (Table 2), which must be interpreted in light of the recommendations concerning its use. The vast majority of these hospitalizations (98.4%) were in conventional wards (only two patients were in intensive care units) for a median hospital stay of 7 [5; 14] days.

There were significantly fewer patients who received tacrolimus in the NTZ group (Table 3). Steroids dose was significantly higher in the FUM group compared to the others (*p* = 0.003). Nearly half of patients (n = 60/121, 49.6%) had significant tacrolimus trough levels (>10 ng/L) at the time of microsporidiosis diagnosis (Table 3).

**TABLE 3:** Management and follow-up.

	All patients (n = 154)	MIT (n = 64)	Fumagillin (n = 54)	Nitazoxanide (n = 36)	p
Immunosuppressive treatment at day 0					
Tacrolimus	134 (87.0)	58 (90.6)	50 (92.6)	26 (72.2)	0.02
Trough levels (ng/L) (n = 121)	10.0 [6.6; 14.7]	9.9 [7.1; 13.9]	9.8 [5.5; 14.7]	11.2 [6.5; 14.8]	0.85
Trough levels >10 ng/L	60/121 (49.6)	25/52 (48.1)	24/48 (50.0)	11/21 (52.4)	0.94
Mycophenolate mofetil	128 (83.1)	51 (79.7)	49 (90.7)	28 (77.8)	0.17
Dose (g/day) (n = 124)	1.0 [1.0; 1.5]	1.0 [1.0; 1.0]	1.0 [1.0; 1.5]	1.0 [0.8; 1.5]	0.63
Steroids	105 (68.2)	45 (70.3)	38 (70.4)	22 (61.1)	0.58
Dose (mg/day) (n = 103)	5.0 [5.0; 10.0]	5.0 [5.0; 5.0]	7.5 [5.0; 10.0]	5.0 [5.0; 10.0]	0.003
Everolimus	15 (9.7)	9 (14.1)	2 (3.7)	4 (11.1)	0.14
Dose (mg/day) (n = 15)	2.0 [1.5; 3.0]	2.0 [2.0; 4.0]	2.5 [2.0; 3.0]	1.5 [1.3; 1.8]	0.15
Cyclosporine	14 (9.1)	3 (4.7)	2 (3.7)	9 (25.0)	0.002
Dose (mg/day) (n = 13)	120 [100; 200]	160 [60; 400]	85 [50; 120]	120 [110; 200]	0.41
Azathioprine	5 (3.3)	2 (3.1)	2 (3.7)	1 (2.8)	1.00
Dose (mg/day) (n = 4)	88 [63; 100]	63 [50; 75]	100	100	0.26
Belatacept	4 (2.6)	1 (1.6)	3 (5.6)	0	0.36
Dose (mg/day) (n = 4)	340 [265; 395]	380	300 [230; 410]	—	0.65
Follow-up and outcome					
Clinical remission	134 (87.0)	57 (89.1)	49 (90.7)	28 (77.8)	0.23
Time from first symptoms to clinical remission (days) (n = 76)	10 [5; 21]	8 [5; 15]	14 [7; 24]	9 [5; 25]	0.28
Serum creatinine at month 3 (µmol/L) (n = 126)	144 [115; 203]	137 [110; 195]	159 [116; 203]	139 [117; 209]	0.69
Microsporidia stool monitoring	72 (46.8)	22 (34.4)	36 (66.7)	14 (38.9)	0.08
Stool negativization rate	50/72 (69.4)	13/22 (59.1)	33/36 (91.7)	4/14 (28.6)	0.002
Relapse	10/145 (6.9)	4/57 (7.0)	1/53 (1.9)	5/35 (14.3)	0.13
Organ failure at month 12	1/101 (1.0)	0/50 (0.0)	0/23 (0.0)	1/28 (3.6)	0.51
Death at month 12	1/124 (0.8)	0/50 (0.0)	1/47 (2.1)	0/27 (0.0)	0.60

Data are presented as number of patients (percentages), or median [25th; 75th percentiles]. Comparisons between groups for immunosuppressive treatment at day 0 were made with Chi-squared test or Fisher’s exact test for categorical data, and with Kruskal-Wallis test for quantitative data. Comparisons between groups for outcomes were made with linear or generalized linear mixed models, with the center as random effect. In the first column, “n” is the number of available data when there is missing data. MIT: modification of immunosuppressive treatment.

Median duration of treatment with nitazoxanide and fumagillin was 14 [13; 21] and 14 [12; 14] days, respectively. Regarding nitazoxanide, three (8.3%) patients received less than 7 days of treatment, 17 (47.2%) patients received more than 14 days, and three (8.3%) patients received treatment several times for at least 14 days. Regarding fumagillin, seven (13.0%) patients received 7 days of treatment, and five (9.3%) patients received more than 14 days. The reasons for treatment durations of 7 days, whether for nitazoxanide or fumagillin, are not known.

### Safety and Treatment Interactions

One patient (kidney transplant recipient) (2.8%) treated with nitazoxanide experienced hepatic adverse effects attributed to the treatment, but this did not lead to treatment discontinuation. Regarding patients treated with fumagillin, as expected, thrombocytopenia was reported in 86.8% (n = 33/38) of cases (Supplementary Figure S1). Median nadir platelet count was 70 [40; 124] G/L, and severe (*i.e.*, < 50 G/L) thrombocytopenia was observed in 34.2% (n = 13/38) of patients. Severe thrombocytopenia led to premature stoppage of treatment in four patients, after 10 days of treatment for two of them and 13 days for the other two. Only one patient developed a hemorrhagic event (an intra-alveolar hemorrhage which occurred a few days after stopping fumagillin when the thrombocytopenia worsened). For all patients, thrombocytopenia was reversible several days after stopping treatment. There was no effect of serum creatinine value at initiation of fumagillin treatment on fumagillin tolerance.

No drug–drug interactions were reported. In particular, there were no reported cases of difficulty obtaining the therapeutic target concentrations of IS drugs in the presence of fumagillin or nitazoxanide.

### Follow-Up and Outcome

**FIGURE 2:**
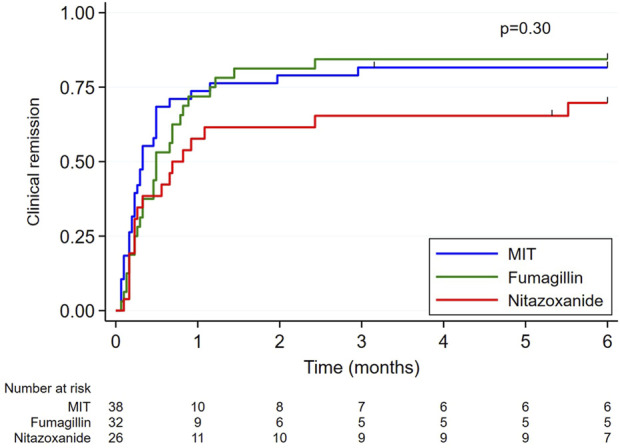
Graphical representation of clinical remission times according to treatment. Clinical remission times (censored data) were estimated by the Kaplan-Meier method and compared between groups using the log-rank test. The analysis was carried out on 96 data, 58 clinical remission times not being specified. MIT: modification of immunosuppressive treatment.

Three months after diagnosis, median serum creatinine value was 144 [115; 203] µmol/L. Symptoms associated with microsporidiosis disappeared for 134 (87.0%) patients, within a median of 10 [5; 20] days following the start of specific treatment and/or modification of IS treatment. Clinical remission rate was 77.8% for the NTZ group, 89.1% for the MIT group, and 90.7% for the FUM group, with no significant between-group differences (Table 3; Figure 2). By adjusting the effect of age, sex and serum creatinine, there is no difference in terms of clinical remission (n = 154; *p* = 0.49). Likewise, no difference was observed by adjusting the effect of age, sex and renal failure (n = 98; *p* = 0.78).

**FIGURE 3:**
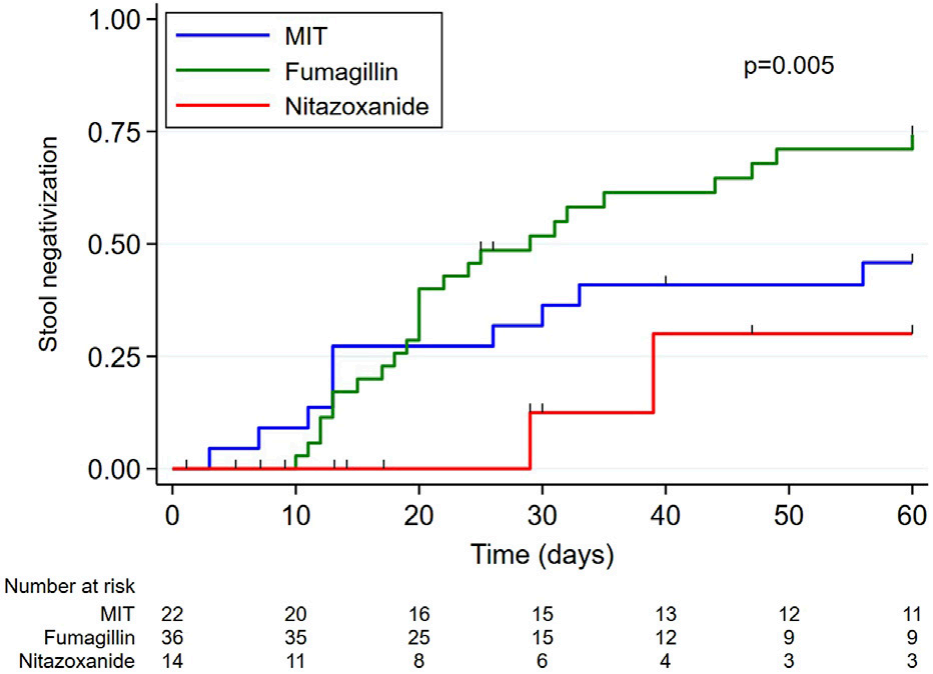
Graphical representation of stool negativization times according to treatment. Times to stool negativization (censored data) were estimated by the Kaplan-Meier method and compared between groups using the log-rank test. MIT: modification of immunosuppressive treatment.

Microsporidia monitoring on patient stools was only carried out in less than half of the patients (46.8%). Stool negativization rate was significantly higher in the FUM group compared to MIT and NTZ groups (91.7% vs. 59.1% vs. 28.6% respectively, *p* = 0.002) (Table 3; Figure 3).

Ten out of 145 patients (6.9%) relapsed. Median time to relapse was 150 [102; 526] days after the first positive-test sample of the first episode. Relapses tended to be more frequent in the NTZ group than in the MIT and FUM groups (14.3% vs. 7.0% vs. 1.9% respectively, *p* = 0.13). All relapsed patients in the NTZ group received at least 14 days of nitazoxanide (two had received nitazoxanide for 30 days). Conversely, the lowest rate of relapses was observed in the FUM group, with only one case (1.9%). Note that this patient received fumagillin only during 7 days and relapsed after 294 days.

### Graft and Patient Survival

Six kidney transplant recipients experienced acute graft rejection during the 3 months following microsporidiosis diagnosis (five MIT and one NTZ). All the rejection cases were attributed to the reduction in IS dose.

One patient, a kidney-pancreas transplant recipient belonging to NTZ group and diagnosed with chronic rejection before the onset of microsporidiosis, presented kidney transplant failure after 5 months.

One kidney transplant recipient in the FUM group died of hemorrhagic stroke at 5 months, with no direct link to microsporidiosis or treatment.

## Discussion

In the context of a fumagillin shortage since several years, we conducted this study to describe management practices and challenges with *E. bieneusi* microsporidiosis in SOT recipients.

More than 40% of the *E. bieneusi* microsporidiosis patients in this study were managed by adapting their IS treatment only, and the outcome was favorable in 89.1% of cases. Interestingly, patients treated with a specific anti-microsporidia drug (fumagillin or nitazoxanide) presented more severe clinical (*i.e.*, hospitalization rate, tri-therapy immunosuppressant regimen) and/or biological (*i.e.*, severe acute renal failure AKIN 3) status at the time of microsporidiosis diagnosis. Note that all six graft rejections identified in the 3 months following microsporidiosis were attributed to IS reduction as infection management strategy.

Clinical remission rates were not significantly different between groups but nitazoxanide tended to be the least effective strategy, with a clinical remission rate of 77.8%. Interestingly, nearly half of patients treated with nitazoxanide received more than 14 days of treatment, suggesting that 14 days may be too short. Dosage and duration of nitazoxanide treatment have not been defined for microsporidiosis, but previously published cases reported success following treatment with 1,000 mg nitazoxanide twice daily for 60 days, or 500 mg twice daily usually for 14 days but also up to more than a year [7–11]. Fumagillin was not more effective than IS management alone in obtaining clinical remission (90.7% and 89.1%, respectively). However, fumagillin was the most effective treatment for achieving stool negativization (91.7% vs. 28.6% for nitazoxanide alone and 59.1% for IS management alone). Nitazoxanide treatment also had the highest relapse rate, at 14.3%, compared to 1.9% for fumagillin and 7.0% for IS management alone, but the difference was not statistically significant. The only relapse after fumagillin treatment was associated with premature discontinuation of treatment, whereas fumagillin was effective for the other six patients who were also prematurely discontinued. This raises the question of the necessary duration of treatment in SOT recipients, as the recommended duration of 14 days had initially been defined from a cohort consisting mainly of HIV patients [4]. Finally, note that there were no graft rejections reported in fumagillin-group patients. Importantly, these comparisons between therapeutic strategies must also take into account that patients of each treatment group have different baseline characteristics, which introduces potential confounding factors and may influence the success or failure of treatment. However, by adjusting the effect of age, sex and renal failure, there is no difference in terms of clinical remission.

Interestingly, at the time of microsporidiosis diagnosis, almost 50% of patients had elevated tacrolimus levels (above 10 ng/mL). Over-dosing of tacrolimus was previously described in patients with infectious diarrhea [16, 17], including microsporidiosis [2]. This phenomenon is caused by the impaired function of P-glycoprotein efflux proteins, leading to an increase in intestinal absorption of tacrolimus between 90 and 360 min after intake [18].

Even though fumagillin proved very effective against *E. bieneusi*, its side effects and contraindications warrant caution. The tolerance and effectiveness of fumagillin have not been established for patients over 65 years old [19]. Likewise, serum creatinine higher than 175 μmol/L contraindicates the use of fumagillin, although there is apparently nothing in the summary of characteristics of the medicinal product to justify this point [19]. In our retrospective study, 18 fumagillin-group patients were between 65 and 83 years old and did not develop more adverse events than their younger counterparts [3/33 (9.1%) for patients under 65 and 1/18 (5.6%) for patients 65 and over]. Interestingly, median serum creatinine at initiation of fumagillin treatment was 191 [129; 278] µmol/L (maximum serum creatinine was as high as 838 μmol/L). Thrombocytopenia was observed in 86.8% of cases after 14 days of treatment, and as previously described by Maillard et *al*., severe thrombocytopenia occurred more frequently in patients with an initial platelet count below 200 G/L (Supplementary Figure S1) [6].

This study has certain limitations, mainly due to its retrospective design. Some results may be due to center-dependent effects, for example, fumagillin co-prescribed with high-dose steroids. The absence of systematic microsporidia follow-up (particularly at the end of the treatment) was also a limitation to confirming stool negativization after clinical remission, and it underscores the lack of clear recommendations on microsporidia follow-up of patients during the course of treatment. Such follow-up would also be informative to evaluate whether a fumagillin treatment duration of 14 days is necessary to achieve stool negativization in SOT patients. This lack of microsporidia follow-up further complexified the diagnosis of relapse: as no samples from the initial infection and relapse were available to compare the strains, we were unable to formally distinguish between a relapse and a new infection event on the one hand, but also between a relapse and a treatment failure on the other hand.

In conclusion, *E. bieneusi* infections in SOT recipients remain life-threatening diseases, as all cases of acute graft rejection in our cohort were attributed to the reduction in IS treatment required to manage microsporidiosis. However, adaptation of IS treatment alone was as effective as nitazoxanide treatment for the management of *E. bieneusi* infection. Fumagillin was particularly effective for achieve clinical remission and fast microsporidia clearance with minimal risk of relapse. Moreover, no major fumagillin-related adverse events were observed in patients over 65 years old or with serum creatinine above 175 μmol/L. The unavailability of fumagillin remains a problem for treatment, particularly for patients for whom it is not possible to modify IS treatment or with the most severe clinical presentation.

## Data Availability

The raw data supporting the conclusions of this article will be made available by the authors, without undue reservation.
