# Supplementary material for: Fumagillin Shortage: How to Treat Enterocytozoon bieneusi Microsporidiosis in Solid Organ Transplant Recipients in 2024?
Source: Transpl Int. 2024 Dec 12;37:13518. doi: 10.3389/ti.2024.13518 (PMC11670256; doi:10.3389/ti.2024.13518)
Supplement: Supplementary file 1 [file DataSheet1.docx]

**Supp Table S1. Infections occurring at the same time as microsporidiosis.**

These concomitant infections concerned 47 patients.

|  | **Infectious agent** | **Number of cases** |
| --- | --- | --- |
| **Pulmonary infections** | |  |
| Bacterial | *Staphylococcus aureus*  *Escherichia coli*  Not identified | 1  1  1 |
| Viral | Severe acute respiratory syndrome coronavirus 2 SARS-CoV-2 | 2 |
| Fungal | *Pneumocystis jirovecii* | 1 |
| **Digestive infections** | |  |
| Bacterial | Enteropathogenic *Escherichia coli* EPEC  *Clostridioides difficile*  *Campylobacter jejuni*  *Helicobacter pylori*  *Salmonella* non Typhi  *Aeromonas hydrophila*  *Aeromonas veronii*  *Vibrio parahaemolyticus*  Enteroaggregative *Escherichia coli* EAEC  *Klebsiella oxytoca*  Not identified | 7  5  3  1  1  1  1  1  1  1  1 |
| Viral | Cytomégalovirus CMV  Norovirus  Rotavirus  Adenovirus | 9  4  2  1 |
| Fungal | *Scedosporium* sp. | 1 |
| **Urinary infections** | |  |
| Bacterial | *Enterobacter* sp.  *Escherichia coli*  Not identified | 1  2  1 |
| **Renal infections** | |  |
| Bacterial | *Escherichia coli*  *Enterococcus faecalis*  *Enterococcus* *faecium*  *Pseudomonas aeruginosa*  Not identified | 3  1  1  1  2 |
| **Systemic infections** | |  |
| Bacterial | *Staphylococcus aureus* | 1 |
| Viral | Cytomegalovirus CMV  Parvovirus B19  Varicelle-zona virus VZV | 3  1  1 |


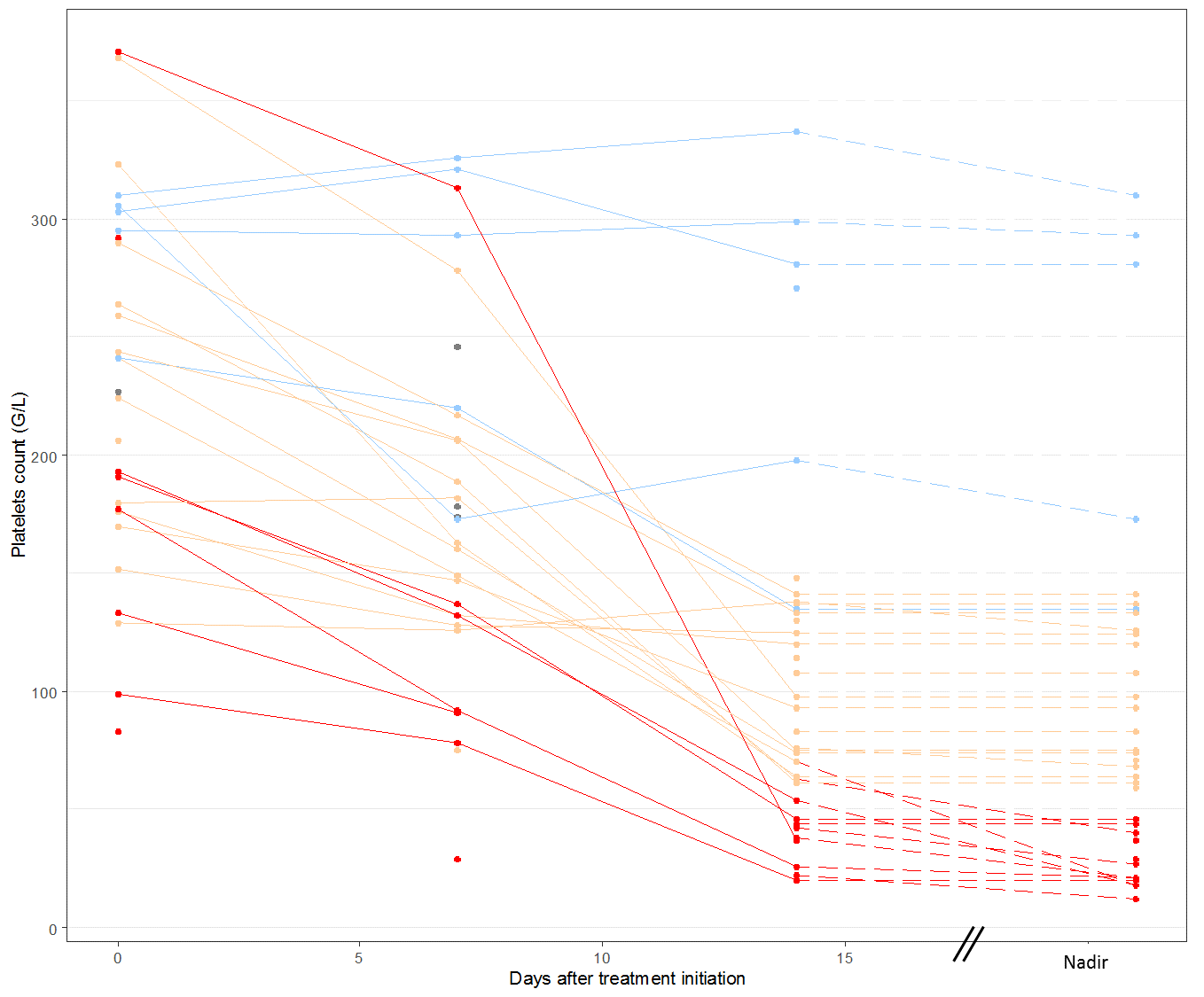


**Supp Figure S1. Graphical representation of platelets count during fumagillin treatment and nadir value.**

*The blue lines represent changes in platelet counts with a nadir value >150 G/L (absence of thrombocytopenia).*

*The orange lines represent changes in platelet counts with a nadir value between 50 and 150 G/L (mild thrombocytopenia).*

*The red lines represent changes in platelet counts with a nadir value <50 G/L (severe thrombocytopenia).*
